# Bis(9-amino­acridinium) bis­(pyridine-2,6-dicarboxyl­ato-κ^3^ *O* ^2^,*N*,*O* ^6^)manganate(II) trihydrate

**DOI:** 10.1107/S1600536811036981

**Published:** 2011-09-30

**Authors:** Hossien Eshtiagh-Hosseini, Masoud Mirzaei, Ehsan Eydizadeh, Zakieh Yousefi, Krešimir Molčanov

**Affiliations:** a Department of Chemistry, Ferdowsi University of Mashhad, 917791436 Mashhad, Iran; b Laboratory of Chemical Crystallography and Biocrystallography, Department of Physical Chemistry, Rudjer Bošković Institute, Bijenička 54, HR-10000 Zagreb, Croatia

## Abstract

The asymmetric unit of the title compound, (C_13_H_11_N_2_)_2_[Mn(C_7_H_3_NO_4_)_2_]·3H_2_O, consists of a discrete mononuclear [Mn(2,6-pydc)_2_]^2−^ anionic complex (2,6-pydc is pyridine-2,6-dicarboxyl­ate) associated with two 9-amino­acridinium counter-ions for neutralization of charge and three uncoordin­ated water mol­ecules. The Mn^II^ atom is six-coordinated by (2,6-pydc)^2−^ anions in a tridentate fashion and is at the centre of a distorted octa­hedron formed by the MnO_4_N_2_ bonding set. In the crystal, various inter­molecular inter­actions between different moieties can be found, such as different kinds of hydrogen bonds, offset or slipped π–π [centroid–centroid distances in the range 3.3704 (12) to 3.8674 (13)Å] and C=O⋯π [3.563 Å] inter­actions, which lead to the formation of a three-dimensional supra­molecular network.

## Related literature

For complexes derived from Mn(II) atoms and pyridine-2,6-dicarboxlic acid, see: Aghabozorg *et al.* (2010 ▶, 2011 ▶). For similar compounds, see: Mirzaei *et al.* (2011 ▶); Derikvand *et al.* (2010 ▶); Eshtiagh-Hosseini, Aghabozorg *et al.* (2010 ▶); Eshtiagh-Hosseini, Alfi *et al.* (2010 ▶); Eshtiagh-Hosseini, Gschwind *et al.* (2010 ▶); Eshtiagh-Hosseini, Yousefi *et al.* (2010 ▶) ; Mei & Wolf (2004 ▶); MacDonald *et al.* (2000 ▶); Aghabozorg *et al.* (2008 ▶).
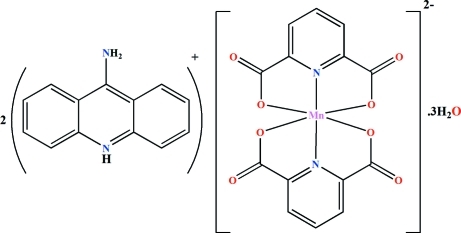


## Experimental

### 

#### Crystal data

(C_13_H_11_N_2_)_2_[Mn(C_7_H_3_NO_4_)_2_]·3H_2_O
*M* *_r_* = 829.67
Triclinic, 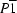

*a* = 10.8202 (4) Å
*b* = 13.5186 (5) Å
*c* = 13.9844 (5) Å
α = 102.351 (3)°
β = 103.466 (3)°
γ = 104.868 (3)°
*V* = 1839.42 (12) Å^3^
*Z* = 2
Cu *K*α radiation
μ = 3.55 mm^−1^
*T* = 293 K
0.20 × 0.12 × 0.10 mm

#### Data collection

Oxford Diffraction Xcalibur Nova diffractometer
Absorption correction: multi-scan (*CrysAlis PRO*; Oxford Diffraction, 2010 ▶) *T*_min_ = 0.538, *T*_max_ = 0.718
17948 measured reflections
7606 independent reflections
6791 reflections with *I* > 2σ(*I*)
*R*_int_ = 0.029

#### Refinement

*R*[*F* ^2^ > 2σ(*F* ^2^)] = 0.038
*wR*(*F* ^2^) = 0.114
*S* = 1.08
7606 reflections
572 parameters
9 restraints
H atoms treated by a mixture of independent and constrained refinement
Δρ_max_ = 0.25 e Å^−3^
Δρ_min_ = −0.31 e Å^−3^

### 

Data collection: *CrysAlis PRO* (Oxford Diffraction, 2010 ▶); cell refinement: *CrysAlis PRO*; data reduction: *CrysAlis PRO*; program(s) used to solve structure: *SHELXS86* (Sheldrick, 2008 ▶); program(s) used to refine structure: *SHELXL97* (Sheldrick, 2008 ▶); molecular graphics: *ORTEP-3 for Windows* (Farrugia, 1997 ▶); software used to prepare material for publication: *WinGX* (Farrugia, 1999 ▶) and *PLATON* (Spek; 2009 ▶).

## Supplementary Material

Crystal structure: contains datablock(s) global, I. DOI: 10.1107/S1600536811036981/om2447sup1.cif

Structure factors: contains datablock(s) I. DOI: 10.1107/S1600536811036981/om2447Isup2.hkl

Additional supplementary materials:  crystallographic information; 3D view; checkCIF report

## Figures and Tables

**Table 1:** Hydrogen-bond geometry (Å, °)

*D*—H⋯*A*	*D*—H	H⋯*A*	*D*⋯*A*	*D*—H⋯*A*
N3—H3*N*⋯O2	0.88 (2)	1.86 (2)	2.736 (2)	178 (2)
N4—H4*A*⋯O4^i^	0.85 (3)	2.22 (3)	2.994 (2)	152 (3)
N4—H4*B*⋯O10^ii^	0.93 (3)	2.01 (3)	2.884 (3)	156 (2)
N5—H5*N*⋯O11	0.84 (3)	1.87 (3)	2.706 (2)	171 (3)
N6—H6*A*⋯O6	0.90 (2)	1.92 (3)	2.801 (2)	164 (2)
N6—H6*B*⋯O1^iii^	0.89 (3)	2.20 (3)	3.029 (2)	155 (3)
O9—H9*A*⋯O4	0.94 (3)	1.89 (3)	2.815 (2)	171 (3)
O9—H9*B*⋯O4^iv^	0.93 (3)	1.93 (3)	2.851 (3)	172 (3)
O10—H10*A*⋯O3^v^	0.92 (2)	2.02 (2)	2.926 (2)	168 (3)
O10—H10*B*⋯O8	0.92 (3)	1.89 (3)	2.805 (3)	175 (4)
O11—H11*A*⋯O6^vi^	0.92 (3)	1.88 (3)	2.790 (3)	170 (4)
O11—H11*B*⋯O9^vii^	0.92 (3)	1.85 (3)	2.756 (3)	169 (2)

Symmetry codes: (i) 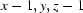; (ii) 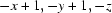; (iii) 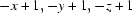; (iv) 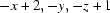; (v) 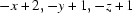; (vi) 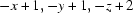; (vii) 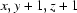.

**Fig. 1.**
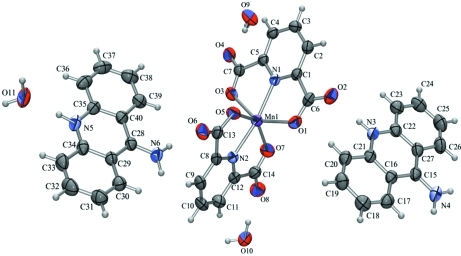
ORTEP view of the title molecule, with the atom-numbering scheme. Displacement ellipsoids are drawn at the 50% probability level.

**Fig. 2.**
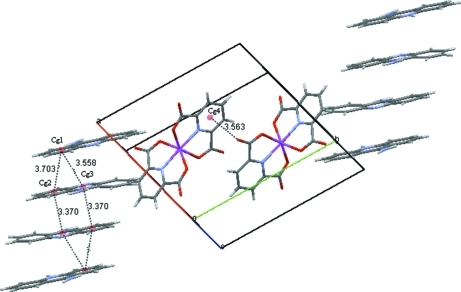
Packing diagram of the title compoud showing extensive π···π stacking interaction between aromatic rings of (9aaH)+ ions and carbonyl···π interactions (Cg1—Cg2= 3.703 Å, Cg1=C16—C17—C18—C19—C20—C21 and Cg2= C34—C35—C36—C37—C39—C40; Cg1—Cg3= 3.515 Å, Cg3=C28—C29—C34—C35—C40—N5; Cg2—Cg3=3.370 Å; Cg4—O= 3.563 Å, Cg4=C8—C9—C10—C11—C12—N2).

## supplementary crystallographic information

### Comment

Acridine derivatives with two benzene rings fused to pyridine are highly fluorescent agents. These compounds are used as topical antiseptics and experimentally as mutagens, intracellular pH indicators and as MALDI matrices (Derikvand *et al.* 2010). Acridine and related derivatives bind to DNA and RNA due to their abilitiy to intercalate. In the viewpoint of crystal engineering, acridine and its 9-amino derivative are very interesting because of their capability for hydrogen bonding *via* N atom of the ring and π-π stacking since they possess three rings (Aghabozorg *et al.* 2010, Mei & Wolf, 2004).

In continuation of our study on proton transfer compounds and their complexes (Mirzaei *et al.*, 2011; Eshtiagh-Hosseini, Aghabozorg *et al.*, 2010; Eshtiagh-Hosseini, Alfi *et al.*, 2010; Eshtiagh-Hosseini, Gschwind *et al.*, 2010; Eshtiagh-Hosseini, Yousefi *et al.*, 2010), here we describe the crystal structure of a new coordination compound based upon Mn^II^ atom, 2,6-pydcH_2_, and 9aa (abbreviation for 9-aminoacridine) fragments. The asymmetric unit of 1 comprises an anionic complex [Mn(2,6-pydc)_2_]^2–^, two monoprotonated (9aaH^+^), and three uncoordinated water molecules (Fig. 1). The Mn^II^ atom is six-coordinated *via* two tridentate (2,6-pydc)^2–^ with polyhedron MnO_4_N_2_ which adopts a distorted octahedral geometry. Indeed, in anionic fragment two rigid (2,6-pydc)^2–^ are almost perpendicular to each other. The geometry, bond distances and angles of title compound are comprable with similar compounds in reported litratures (Aghabozorg *et al.*, 2008; MacDonald *et al.* 2000) It is interesting to point out that in the crystal structure of 1, two cationic fragments participate in different H-bonds, that is, one of them takes part in three H-bonds *via* NH_2_ group and an N atom located in the ring with anionic complex fragments and water molecule, while, the other partakes in H-bond with a water molecule and an anionic moiety *via* with NH_2_ group and with another complex *via* N atom of ring. Also, there is a H-bond pattern with graph set *R*^4^_2_(8) created by two water molecules and two complex fragments. Alongside different H-bonds, π-π stacking interactions play an important role in the stability of 1. As claimed before, 9aa can establish several π-π stacking interactions, and this point is evident in this structure, as well as the intermolecular π-π interaction which occurs between the two symmetry-related anionic fragments (2,6-pydc)^2–^. Distances between centroids of aromatic rings range from 3.3704 (12)Å to 3.8674 (13)Å (*Cg*1—*Cg*2= 3.703 Å, *Cg*1=C16—C17—C18—C19—C20—C21 and *Cg*2= C34—C35—C36—C37—C39—C40; *Cg*1—*Cg*3= 3.515 Å, *Cg*3=C28—C29—C34—C35—C40—N5; *Cg*2—*Cg*3=3.370 Å) as calculated by *PLATON* (Spek, 2009). In addition to these intermolecular interactions, some weak interactions such as C=O···π (*Cg*4—O= 3.563 Å, *Cg*4=C8—C9—C10—C11—C12—N2), aid to construct a three-dimensional supramolecular network (Fig. 2).

### Experimental

To an aqeous solution of pydcH_2_ (0.0167 g, 0.1 mmol) a solution of 9a-*ac* (0.02 g,0.1 mmol) in methanol was added dropwise, then a soution of MnCl_2_.2H_2_O(0.0167 g, 0.1 mmol)) in water was added and the resultant solution was heated and stirred for 3 hrs at 60 °C. The yellow crystals were obtained by slow evaporation at room temprature after 3 days.

### Refinement

The structure was refined using the full-matrix least-squares refinement included in the SHELXL-97 (Sheldrick, 2008). All non-hydrogen atoms were refined anisotropically. Hydrogen atoms bound to N and O atoms were located from the difference Fourier map and refined as free entities; O–H bonds were restrained to 0.95 (2) Å and H–H distances to 1.50 (4) Å. Hydrogen atoms bond to carbon atoms placed according to their geometrical environment and refined using a riding model with C–H distance 0.93 Å and *U*_iso_(H) = 1.2*U*_eq_(C).

### Crystal data

**Table:**

(C_13_H_11_N_2_)_2_[Mn(C_7_H_3_NO_4_)_2_]·3H_2_O	*Z* = 2
*M**_r_* = 829.67	*F*(000) = 858
Triclinic, *P*1	*D*_x_ = 1.498 Mg m^−^^3^
*a* = 10.8202 (4) Å	Cu *K*α radiation, λ = 1.54184 Å
*b* = 13.5186 (5) Å	Cell parameters from 11391 reflections
*c* = 13.9844 (5) Å	θ = 3.4–75.9°
α = 102.351 (3)°	µ = 3.55 mm^−^^1^
β = 103.466 (3)°	*T* = 293 K
γ = 104.868 (3)°	Prism, yellow
*V* = 1839.42 (12) Å^3^	0.20 × 0.12 × 0.10 mm

### Data collection

**Table:**

Oxford Diffraction Xcalibur Nova diffractometer	7606 independent reflections
Radiation source: Enhance (Cu) X-ray Source	6791 reflections with *I* > 2σ(*I*)
graphite	*R*_int_ = 0.029
Detector resolution: 10.4323 pixels mm^-1^	θ_max_ = 76.1°, θ_min_ = 3.4°
ω scans	*h* = −13→13
Absorption correction: multi-scan (*CrysAlis PRO*; Oxford Diffraction, 2010)	*k* = −17→16
*T*_min_ = 0.538, *T*_max_ = 0.718	*l* = −17→12
17948 measured reflections	

### Refinement

**Table:**

Refinement on *F*^2^	H atoms treated by a mixture of independent and constrained refinement
Least-squares matrix: full	*w* = 1/[σ^2^(*F*_o_^2^) + (0.0654*P*)^2^ + 0.2228*P*] where *P* = (*F*_o_^2^ + 2*F*_c_^2^)/3
*R*[*F*^2^ > 2σ(*F*^2^)] = 0.038	(Δ/σ)_max_ < 0.001
*wR*(*F*^2^) = 0.114	Δρ_max_ = 0.25 e Å^−^^3^
*S* = 1.08	Δρ_min_ = −0.31 e Å^−^^3^
7606 reflections	Extinction correction: *SHELXL97* (Sheldrick, 2008), Fc^*^=kFc[1+0.001xFc^2^λ^3^/sin(2θ)]^-1/4^
572 parameters	Extinction coefficient: 0.00077 (19)
9 restraints	

### Special details

**Table:**

Geometry. All esds (except the esd in the dihedral angle between two l.s. planes) are estimated using the full covariance matrix. The cell esds are taken into account individually in the estimation of esds in distances, angles and torsion angles; correlations between esds in cell parameters are only used when they are defined by crystal symmetry. An approximate (isotropic) treatment of cell esds is used for estimating esds involving l.s. planes.

### Fractional atomic coordinates and isotropic or equivalent isotropic displacement parameters (Å^2^)

**Table:**

	*x*	*y*	*z*	*U*_iso_*/*U*_eq_	
Mn1	0.64063 (3)	0.25673 (2)	0.40959 (2)	0.04394 (10)	
O1	0.44381 (15)	0.20160 (11)	0.28655 (10)	0.0518 (3)	
O2	0.26457 (17)	0.05818 (15)	0.19184 (12)	0.0669 (4)	
O3	0.79344 (15)	0.20184 (11)	0.50812 (11)	0.0535 (3)	
O4	0.84704 (14)	0.06026 (11)	0.53782 (11)	0.0529 (3)	
N1	0.56149 (14)	0.08499 (11)	0.37675 (10)	0.0360 (3)	
C1	0.43716 (17)	0.03411 (14)	0.31346 (11)	0.0377 (3)	
C2	0.37476 (18)	−0.07366 (15)	0.29815 (13)	0.0451 (4)	
H2	0.2881	−0.1084	0.2535	0.054*	
C3	0.44423 (19)	−0.12868 (14)	0.35074 (14)	0.0447 (4)	
H3	0.4042	−0.2009	0.3422	0.054*	
C4	0.57380 (18)	−0.07533 (13)	0.41616 (12)	0.0404 (3)	
H4	0.6221	−0.1111	0.4517	0.048*	
C5	0.62957 (16)	0.03236 (13)	0.42732 (11)	0.0356 (3)	
C6	0.37496 (19)	0.10379 (16)	0.25923 (13)	0.0444 (4)	
C7	0.76853 (18)	0.10354 (14)	0.49707 (12)	0.0407 (3)	
O5	0.54528 (17)	0.30295 (10)	0.52945 (11)	0.0543 (3)	
O6	0.52901 (17)	0.43798 (12)	0.64314 (11)	0.0581 (4)	
O7	0.76616 (18)	0.31403 (12)	0.31869 (11)	0.0609 (4)	
O8	0.91391 (19)	0.45506 (14)	0.30369 (13)	0.0657 (4)	
N2	0.71790 (16)	0.42813 (11)	0.47040 (10)	0.0416 (3)	
C8	0.67712 (19)	0.47705 (14)	0.54468 (12)	0.0425 (4)	
C9	0.7249 (2)	0.58709 (15)	0.58508 (14)	0.0528 (4)	
H9	0.6945	0.6213	0.6354	0.063*	
C10	0.8191 (3)	0.64496 (16)	0.54865 (17)	0.0605 (5)	
H10	0.8542	0.719	0.5757	0.073*	
C11	0.8613 (2)	0.59309 (16)	0.47198 (16)	0.0567 (5)	
H11	0.9251	0.6313	0.4474	0.068*	
C12	0.8062 (2)	0.48295 (14)	0.43303 (13)	0.0453 (4)	
C13	0.5753 (2)	0.40030 (14)	0.57584 (12)	0.0435 (4)	
C14	0.8333 (2)	0.41252 (16)	0.34409 (14)	0.0493 (4)	
N3	0.14305 (15)	0.10470 (12)	0.02023 (11)	0.0411 (3)	
H3N	0.181 (2)	0.0906 (19)	0.0761 (19)	0.050 (6)*	
N4	−0.01292 (19)	0.18331 (17)	−0.24082 (13)	0.0553 (4)	
H4A	−0.029 (3)	0.141 (2)	−0.300 (2)	0.065 (7)*	
H4B	−0.029 (3)	0.248 (2)	−0.236 (2)	0.073 (8)*	
C15	0.03526 (17)	0.15624 (15)	−0.15714 (13)	0.0428 (4)	
C16	0.05018 (16)	0.22265 (14)	−0.05743 (13)	0.0397 (3)	
C17	0.01312 (18)	0.31667 (16)	−0.04250 (15)	0.0475 (4)	
H17	−0.0232	0.3373	−0.0991	0.057*	
C18	0.0302 (2)	0.37710 (17)	0.05400 (17)	0.0525 (4)	
H18	0.0065	0.4391	0.0627	0.063*	
C19	0.0832 (2)	0.34669 (18)	0.14030 (15)	0.0539 (4)	
H19	0.0924	0.3879	0.2056	0.065*	
C20	0.12132 (19)	0.25721 (16)	0.12938 (14)	0.0468 (4)	
H20	0.1576	0.238	0.187	0.056*	
C21	0.10539 (16)	0.19399 (14)	0.03001 (12)	0.0389 (3)	
C22	0.12958 (16)	0.03901 (14)	−0.07291 (13)	0.0414 (3)	
C23	0.1714 (2)	−0.05174 (16)	−0.07770 (16)	0.0500 (4)	
H23	0.2069	−0.0672	−0.0175	0.06*	
C24	0.1597 (2)	−0.11733 (17)	−0.17090 (18)	0.0589 (5)	
H24	0.1881	−0.177	−0.1737	0.071*	
C25	0.1055 (2)	−0.09545 (19)	−0.26169 (17)	0.0628 (5)	
H25	0.0982	−0.1406	−0.3246	0.075*	
C26	0.0634 (2)	−0.00866 (19)	−0.25909 (15)	0.0586 (5)	
H26	0.0271	0.0046	−0.3204	0.07*	
C27	0.07390 (17)	0.06234 (15)	−0.16393 (13)	0.0445 (4)	
N5	0.66357 (16)	0.67352 (14)	1.11671 (11)	0.0465 (3)	
H5N	0.677 (3)	0.680 (2)	1.180 (2)	0.073 (8)*	
N6	0.54690 (18)	0.61190 (14)	0.80226 (11)	0.0475 (3)	
H6A	0.556 (2)	0.5560 (19)	0.7592 (19)	0.053 (6)*	
H6B	0.523 (3)	0.661 (2)	0.7753 (19)	0.060 (7)*	
C28	0.58159 (16)	0.62845 (13)	0.90294 (12)	0.0375 (3)	
C29	0.56236 (17)	0.71798 (13)	0.96725 (12)	0.0393 (3)	
C30	0.4940 (2)	0.78319 (15)	0.92699 (14)	0.0466 (4)	
H30	0.4611	0.7694	0.8562	0.056*	
C31	0.4756 (2)	0.86606 (17)	0.99064 (18)	0.0569 (5)	
H31	0.4295	0.9079	0.963	0.068*	
C32	0.5257 (3)	0.88850 (18)	1.09755 (18)	0.0627 (6)	
H32	0.5154	0.9468	1.1403	0.075*	
C33	0.5889 (2)	0.82645 (17)	1.13944 (15)	0.0559 (5)	
H33	0.6208	0.8416	1.2105	0.067*	
C34	0.60641 (17)	0.73838 (15)	1.07472 (13)	0.0424 (4)	
C35	0.67653 (16)	0.58457 (15)	1.05882 (13)	0.0424 (4)	
C36	0.73090 (19)	0.51809 (18)	1.10862 (17)	0.0542 (5)	
H36	0.7585	0.5358	1.18	0.065*	
C37	0.7427 (2)	0.42785 (19)	1.05168 (19)	0.0594 (5)	
H37	0.7766	0.3832	1.0846	0.071*	
C38	0.7046 (2)	0.40105 (17)	0.94423 (19)	0.0584 (5)	
H38	0.7144	0.3395	0.9065	0.07*	
C39	0.65279 (19)	0.46540 (15)	0.89438 (15)	0.0476 (4)	
H39	0.6283	0.4474	0.823	0.057*	
C40	0.63637 (16)	0.55866 (14)	0.95048 (13)	0.0396 (3)	
O9	0.88560 (18)	−0.13804 (14)	0.47029 (16)	0.0717 (4)	
H9A	0.866 (3)	−0.0741 (19)	0.486 (3)	0.096 (11)*	
H9B	0.971 (2)	−0.119 (3)	0.463 (3)	0.103 (12)*	
O10	1.0899 (2)	0.63986 (16)	0.28914 (14)	0.0766 (5)	
H10A	1.135 (3)	0.684 (2)	0.3551 (16)	0.087 (10)*	
H10B	1.036 (3)	0.580 (2)	0.298 (3)	0.114 (13)*	
O11	0.6811 (2)	0.69942 (17)	1.31723 (11)	0.0742 (5)	
H11A	0.618 (3)	0.657 (2)	1.338 (3)	0.100 (11)*	
H11B	0.744 (3)	0.750 (2)	1.3740 (19)	0.089 (10)*	

### Atomic displacement parameters (Å^2^)

**Table:**

	*U*^11^	*U*^22^	*U*^33^	*U*^12^	*U*^13^	*U*^23^
Mn1	0.0648 (2)	0.03080 (15)	0.03618 (15)	0.01615 (12)	0.01555 (12)	0.00863 (10)
O1	0.0674 (8)	0.0479 (7)	0.0453 (7)	0.0261 (6)	0.0124 (6)	0.0202 (6)
O2	0.0601 (9)	0.0752 (10)	0.0585 (8)	0.0199 (8)	−0.0034 (7)	0.0314 (8)
O3	0.0560 (8)	0.0393 (7)	0.0511 (7)	0.0095 (6)	0.0022 (6)	0.0079 (5)
O4	0.0479 (7)	0.0520 (7)	0.0527 (7)	0.0204 (6)	0.0020 (6)	0.0123 (6)
N1	0.0452 (7)	0.0343 (6)	0.0295 (6)	0.0150 (5)	0.0110 (5)	0.0090 (5)
C1	0.0442 (8)	0.0426 (8)	0.0285 (7)	0.0162 (7)	0.0117 (6)	0.0118 (6)
C2	0.0437 (9)	0.0473 (9)	0.0385 (8)	0.0089 (7)	0.0084 (7)	0.0120 (7)
C3	0.0532 (10)	0.0353 (8)	0.0447 (9)	0.0101 (7)	0.0160 (7)	0.0140 (7)
C4	0.0518 (9)	0.0385 (8)	0.0370 (8)	0.0207 (7)	0.0147 (7)	0.0146 (6)
C5	0.0426 (8)	0.0364 (8)	0.0303 (7)	0.0162 (6)	0.0126 (6)	0.0091 (6)
C6	0.0491 (9)	0.0548 (10)	0.0344 (7)	0.0223 (8)	0.0115 (7)	0.0180 (7)
C7	0.0457 (9)	0.0409 (8)	0.0348 (7)	0.0157 (7)	0.0103 (6)	0.0091 (6)
O5	0.0814 (10)	0.0353 (6)	0.0494 (7)	0.0152 (6)	0.0312 (7)	0.0112 (5)
O6	0.0752 (9)	0.0496 (8)	0.0451 (7)	0.0153 (7)	0.0266 (7)	0.0016 (6)
O7	0.0890 (11)	0.0468 (7)	0.0525 (8)	0.0196 (7)	0.0377 (8)	0.0110 (6)
O8	0.0794 (10)	0.0665 (10)	0.0610 (9)	0.0203 (8)	0.0370 (8)	0.0254 (7)
N2	0.0566 (8)	0.0335 (7)	0.0335 (6)	0.0140 (6)	0.0127 (6)	0.0090 (5)
C8	0.0561 (10)	0.0368 (8)	0.0304 (7)	0.0147 (7)	0.0081 (6)	0.0067 (6)
C9	0.0733 (13)	0.0380 (9)	0.0383 (8)	0.0150 (8)	0.0125 (8)	0.0021 (7)
C10	0.0792 (14)	0.0332 (9)	0.0544 (11)	0.0049 (9)	0.0154 (10)	0.0046 (8)
C11	0.0662 (12)	0.0426 (10)	0.0539 (11)	0.0062 (9)	0.0169 (9)	0.0143 (8)
C12	0.0567 (10)	0.0401 (9)	0.0375 (8)	0.0130 (7)	0.0112 (7)	0.0147 (7)
C13	0.0600 (10)	0.0402 (9)	0.0304 (7)	0.0182 (7)	0.0130 (7)	0.0089 (6)
C14	0.0649 (11)	0.0473 (10)	0.0409 (9)	0.0198 (8)	0.0193 (8)	0.0177 (7)
N3	0.0403 (7)	0.0457 (8)	0.0354 (7)	0.0127 (6)	0.0078 (5)	0.0137 (6)
N4	0.0621 (10)	0.0606 (10)	0.0367 (8)	0.0191 (8)	0.0042 (7)	0.0139 (7)
C15	0.0351 (8)	0.0483 (9)	0.0374 (8)	0.0065 (7)	0.0050 (6)	0.0123 (7)
C16	0.0316 (7)	0.0459 (9)	0.0396 (8)	0.0097 (6)	0.0087 (6)	0.0144 (7)
C17	0.0403 (8)	0.0532 (10)	0.0501 (9)	0.0169 (7)	0.0096 (7)	0.0198 (8)
C18	0.0466 (10)	0.0519 (10)	0.0625 (11)	0.0214 (8)	0.0195 (8)	0.0137 (9)
C19	0.0554 (11)	0.0572 (11)	0.0465 (9)	0.0188 (9)	0.0175 (8)	0.0066 (8)
C20	0.0484 (9)	0.0531 (10)	0.0375 (8)	0.0139 (8)	0.0130 (7)	0.0131 (7)
C21	0.0326 (7)	0.0431 (8)	0.0385 (8)	0.0083 (6)	0.0102 (6)	0.0125 (7)
C22	0.0359 (8)	0.0415 (8)	0.0418 (8)	0.0070 (6)	0.0107 (6)	0.0098 (7)
C23	0.0471 (9)	0.0464 (10)	0.0556 (10)	0.0140 (8)	0.0146 (8)	0.0152 (8)
C24	0.0576 (11)	0.0439 (10)	0.0700 (13)	0.0145 (9)	0.0218 (10)	0.0063 (9)
C25	0.0663 (13)	0.0557 (12)	0.0523 (11)	0.0120 (10)	0.0180 (9)	−0.0030 (9)
C26	0.0613 (12)	0.0601 (12)	0.0390 (9)	0.0110 (9)	0.0072 (8)	0.0027 (8)
C27	0.0390 (8)	0.0477 (9)	0.0383 (8)	0.0067 (7)	0.0080 (6)	0.0083 (7)
N5	0.0428 (7)	0.0589 (9)	0.0324 (7)	0.0093 (7)	0.0104 (6)	0.0122 (6)
N6	0.0635 (10)	0.0470 (8)	0.0335 (7)	0.0255 (7)	0.0120 (6)	0.0086 (6)
C28	0.0350 (7)	0.0380 (8)	0.0348 (7)	0.0078 (6)	0.0097 (6)	0.0074 (6)
C29	0.0392 (8)	0.0391 (8)	0.0360 (8)	0.0085 (6)	0.0124 (6)	0.0074 (6)
C30	0.0515 (10)	0.0445 (9)	0.0450 (9)	0.0162 (8)	0.0169 (7)	0.0119 (7)
C31	0.0651 (12)	0.0478 (10)	0.0647 (12)	0.0238 (9)	0.0276 (10)	0.0147 (9)
C32	0.0794 (15)	0.0491 (11)	0.0640 (12)	0.0218 (10)	0.0390 (11)	0.0054 (9)
C33	0.0649 (12)	0.0561 (11)	0.0404 (9)	0.0106 (9)	0.0237 (8)	0.0037 (8)
C34	0.0385 (8)	0.0474 (9)	0.0356 (8)	0.0063 (7)	0.0142 (6)	0.0069 (7)
C35	0.0317 (7)	0.0504 (9)	0.0428 (8)	0.0067 (6)	0.0102 (6)	0.0183 (7)
C36	0.0397 (9)	0.0676 (13)	0.0569 (11)	0.0109 (8)	0.0109 (8)	0.0333 (10)
C37	0.0427 (9)	0.0623 (12)	0.0828 (14)	0.0169 (9)	0.0170 (9)	0.0431 (11)
C38	0.0491 (10)	0.0470 (10)	0.0811 (14)	0.0169 (8)	0.0191 (10)	0.0219 (10)
C39	0.0438 (9)	0.0434 (9)	0.0532 (10)	0.0139 (7)	0.0122 (7)	0.0126 (8)
C40	0.0341 (7)	0.0402 (8)	0.0404 (8)	0.0076 (6)	0.0092 (6)	0.0111 (6)
O9	0.0588 (9)	0.0509 (9)	0.0911 (12)	0.0094 (7)	0.0181 (8)	0.0069 (8)
O10	0.0891 (13)	0.0703 (11)	0.0565 (9)	0.0100 (9)	0.0071 (8)	0.0260 (8)
O11	0.0774 (11)	0.0874 (12)	0.0363 (7)	−0.0017 (9)	0.0132 (7)	0.0133 (7)

### Geometric parameters (Å, °)

**Table:**

Mn1—N2	2.1501 (14)	C18—H18	0.93
Mn1—N1	2.1622 (14)	C19—C20	1.365 (3)
Mn1—O7	2.1786 (15)	C19—H19	0.93
Mn1—O5	2.2254 (13)	C20—C21	1.413 (2)
Mn1—O1	2.2319 (15)	C20—H20	0.93
Mn1—O3	2.2780 (14)	C22—C23	1.406 (3)
O1—C6	1.263 (2)	C22—C27	1.412 (3)
O2—C6	1.243 (2)	C23—C24	1.368 (3)
O3—C7	1.254 (2)	C23—H23	0.93
O4—C7	1.249 (2)	C24—C25	1.395 (4)
N1—C1	1.333 (2)	C24—H24	0.93
N1—C5	1.338 (2)	C25—C26	1.360 (4)
C1—C2	1.385 (2)	C25—H25	0.93
C1—C6	1.519 (2)	C26—C27	1.425 (3)
C2—C3	1.387 (3)	C26—H26	0.93
C2—H2	0.93	N5—C34	1.351 (3)
C3—C4	1.387 (3)	N5—C35	1.358 (3)
C3—H3	0.93	N5—H5N	0.84 (3)
C4—C5	1.384 (2)	N6—C28	1.323 (2)
C4—H4	0.93	N6—H6A	0.90 (2)
C5—C7	1.520 (2)	N6—H6B	0.90 (3)
O5—C13	1.255 (2)	C28—C40	1.434 (2)
O6—C13	1.241 (2)	C28—C29	1.438 (2)
O7—C14	1.268 (3)	C29—C34	1.408 (2)
O8—C14	1.233 (3)	C29—C30	1.413 (3)
N2—C12	1.330 (2)	C30—C31	1.363 (3)
N2—C8	1.334 (2)	C30—H30	0.93
C8—C9	1.382 (3)	C31—C32	1.402 (3)
C8—C13	1.520 (3)	C31—H31	0.93
C9—C10	1.383 (3)	C32—C33	1.354 (4)
C9—H9	0.93	C32—H32	0.93
C10—C11	1.387 (3)	C33—C34	1.416 (3)
C10—H10	0.93	C33—H33	0.93
C11—C12	1.384 (3)	C35—C36	1.410 (3)
C11—H11	0.93	C35—C40	1.412 (2)
C12—C14	1.532 (3)	C36—C37	1.360 (4)
N3—C21	1.360 (2)	C36—H36	0.93
N3—C22	1.363 (2)	C37—C38	1.400 (3)
N3—H3N	0.88 (2)	C37—H37	0.93
N4—C15	1.331 (2)	C38—C39	1.373 (3)
N4—H4A	0.85 (3)	C38—H38	0.93
N4—H4B	0.93 (3)	C39—C40	1.412 (3)
C15—C27	1.426 (3)	C39—H39	0.93
C15—C16	1.436 (2)	O9—H9A	0.933 (18)
C16—C21	1.408 (2)	O9—H9B	0.926 (18)
C16—C17	1.417 (3)	O10—H10A	0.924 (18)
C17—C18	1.363 (3)	O10—H10B	0.922 (18)
C17—H17	0.93	O11—H11A	0.923 (18)
C18—C19	1.402 (3)	O11—H11B	0.914 (18)
			
N2—Mn1—N1	169.44 (5)	C18—C17—H17	119.7
N2—Mn1—O7	73.33 (5)	C16—C17—H17	119.7
N1—Mn1—O7	115.96 (5)	C17—C18—C19	120.64 (19)
N2—Mn1—O5	72.69 (5)	C17—C18—H18	119.7
N1—Mn1—O5	98.33 (5)	C19—C18—H18	119.7
O7—Mn1—O5	145.68 (5)	C20—C19—C18	120.60 (18)
N2—Mn1—O1	112.02 (5)	C20—C19—H19	119.7
N1—Mn1—O1	72.97 (5)	C18—C19—H19	119.7
O7—Mn1—O1	97.13 (6)	C19—C20—C21	119.58 (18)
O5—Mn1—O1	91.08 (6)	C19—C20—H20	120.2
N2—Mn1—O3	103.37 (5)	C21—C20—H20	120.2
N1—Mn1—O3	71.55 (5)	N3—C21—C16	120.66 (15)
O7—Mn1—O3	96.10 (6)	N3—C21—C20	118.95 (16)
O5—Mn1—O3	96.27 (6)	C16—C21—C20	120.38 (17)
O1—Mn1—O3	144.44 (5)	N3—C22—C23	119.67 (17)
C6—O1—Mn1	118.13 (11)	N3—C22—C27	119.93 (17)
C7—O3—Mn1	118.29 (11)	C23—C22—C27	120.40 (17)
C1—N1—C5	120.32 (14)	C24—C23—C22	120.0 (2)
C1—N1—Mn1	118.51 (11)	C24—C23—H23	120
C5—N1—Mn1	120.76 (11)	C22—C23—H23	120
N1—C1—C2	121.49 (15)	C23—C24—C25	120.5 (2)
N1—C1—C6	114.05 (15)	C23—C24—H24	119.7
C2—C1—C6	124.43 (16)	C25—C24—H24	119.7
C1—C2—C3	118.59 (16)	C26—C25—C24	120.59 (19)
C1—C2—H2	120.7	C26—C25—H25	119.7
C3—C2—H2	120.7	C24—C25—H25	119.7
C2—C3—C4	119.62 (16)	C25—C26—C27	121.0 (2)
C2—C3—H3	120.2	C25—C26—H26	119.5
C4—C3—H3	120.2	C27—C26—H26	119.5
C5—C4—C3	118.45 (15)	C22—C27—C26	117.47 (19)
C5—C4—H4	120.8	C22—C27—C15	119.44 (16)
C3—C4—H4	120.8	C26—C27—C15	123.06 (18)
N1—C5—C4	121.53 (15)	C34—N5—C35	122.46 (15)
N1—C5—C7	112.96 (14)	C34—N5—H5N	120 (2)
C4—C5—C7	125.51 (15)	C35—N5—H5N	117 (2)
O2—C6—O1	127.50 (17)	C28—N6—H6A	122.2 (15)
O2—C6—C1	116.80 (17)	C28—N6—H6B	120.0 (16)
O1—C6—C1	115.68 (15)	H6A—N6—H6B	117 (2)
O4—C7—O3	125.91 (17)	N6—C28—C40	121.98 (16)
O4—C7—C5	118.23 (16)	N6—C28—C29	119.49 (16)
O3—C7—C5	115.85 (15)	C40—C28—C29	118.52 (15)
C13—O5—Mn1	118.71 (12)	C34—C29—C30	118.19 (16)
C14—O7—Mn1	119.60 (12)	C34—C29—C28	119.07 (16)
C12—N2—C8	121.52 (16)	C30—C29—C28	122.64 (15)
C12—N2—Mn1	118.88 (12)	C31—C30—C29	120.82 (18)
C8—N2—Mn1	119.60 (12)	C31—C30—H30	119.6
N2—C8—C9	120.87 (18)	C29—C30—H30	119.6
N2—C8—C13	113.30 (15)	C30—C31—C32	120.3 (2)
C9—C8—C13	125.81 (17)	C30—C31—H31	119.9
C8—C9—C10	118.25 (19)	C32—C31—H31	119.9
C8—C9—H9	120.9	C33—C32—C31	120.89 (19)
C10—C9—H9	120.9	C33—C32—H32	119.6
C9—C10—C11	120.29 (18)	C31—C32—H32	119.6
C9—C10—H10	119.9	C32—C33—C34	119.79 (19)
C11—C10—H10	119.9	C32—C33—H33	120.1
C12—C11—C10	118.25 (19)	C34—C33—H33	120.1
C12—C11—H11	120.9	N5—C34—C29	120.45 (16)
C10—C11—H11	120.9	N5—C34—C33	119.60 (17)
N2—C12—C11	120.78 (18)	C29—C34—C33	119.95 (18)
N2—C12—C14	113.29 (16)	N5—C35—C36	118.89 (17)
C11—C12—C14	125.90 (18)	N5—C35—C40	120.82 (16)
O6—C13—O5	125.91 (18)	C36—C35—C40	120.29 (19)
O6—C13—C8	118.40 (16)	C37—C36—C35	119.61 (19)
O5—C13—C8	115.69 (15)	C37—C36—H36	120.2
O8—C14—O7	126.68 (19)	C35—C36—H36	120.2
O8—C14—C12	118.80 (18)	C36—C37—C38	121.07 (19)
O7—C14—C12	114.51 (16)	C36—C37—H37	119.5
C21—N3—C22	122.48 (15)	C38—C37—H37	119.5
C21—N3—H3N	118.6 (15)	C39—C38—C37	120.2 (2)
C22—N3—H3N	118.9 (15)	C39—C38—H38	119.9
C15—N4—H4A	119.1 (19)	C37—C38—H38	119.9
C15—N4—H4B	121.4 (18)	C38—C39—C40	120.57 (19)
H4A—N4—H4B	119 (2)	C38—C39—H39	119.7
N4—C15—C27	121.37 (17)	C40—C39—H39	119.7
N4—C15—C16	119.93 (18)	C39—C40—C35	118.27 (17)
C27—C15—C16	118.70 (16)	C39—C40—C28	123.25 (16)
C21—C16—C17	118.15 (16)	C35—C40—C28	118.48 (16)
C21—C16—C15	118.76 (16)	H9A—O9—H9B	105 (3)
C17—C16—C15	123.09 (16)	H10A—O10—H10B	104 (3)
C18—C17—C16	120.63 (18)	H11A—O11—H11B	108 (3)
			
N2—Mn1—O1—C6	−165.89 (13)	Mn1—O5—C13—O6	178.93 (16)
N1—Mn1—O1—C6	4.23 (13)	Mn1—O5—C13—C8	−0.7 (2)
O7—Mn1—O1—C6	119.25 (13)	N2—C8—C13—O6	−178.68 (17)
O5—Mn1—O1—C6	−94.15 (13)	C9—C8—C13—O6	0.3 (3)
O3—Mn1—O1—C6	8.22 (18)	N2—C8—C13—O5	1.0 (2)
N2—Mn1—O3—C7	173.32 (13)	C9—C8—C13—O5	179.98 (19)
N1—Mn1—O3—C7	2.96 (13)	Mn1—O7—C14—O8	175.59 (18)
O7—Mn1—O3—C7	−112.41 (14)	Mn1—O7—C14—C12	−5.7 (2)
O5—Mn1—O3—C7	99.67 (14)	N2—C12—C14—O8	−179.84 (19)
O1—Mn1—O3—C7	−1.07 (19)	C11—C12—C14—O8	2.1 (3)
N2—Mn1—N1—C1	112.5 (3)	N2—C12—C14—O7	1.3 (3)
O7—Mn1—N1—C1	−97.01 (12)	C11—C12—C14—O7	−176.8 (2)
O5—Mn1—N1—C1	81.29 (12)	N4—C15—C16—C21	−178.00 (17)
O1—Mn1—N1—C1	−7.29 (11)	C27—C15—C16—C21	1.5 (2)
O3—Mn1—N1—C1	175.16 (12)	N4—C15—C16—C17	1.4 (3)
N2—Mn1—N1—C5	−60.2 (4)	C27—C15—C16—C17	−179.12 (16)
O7—Mn1—N1—C5	90.30 (12)	C21—C16—C17—C18	−0.3 (3)
O5—Mn1—N1—C5	−91.40 (12)	C15—C16—C17—C18	−179.62 (17)
O1—Mn1—N1—C5	−179.98 (12)	C16—C17—C18—C19	−0.8 (3)
O3—Mn1—N1—C5	2.47 (11)	C17—C18—C19—C20	1.4 (3)
C5—N1—C1—C2	0.1 (2)	C18—C19—C20—C21	−0.9 (3)
Mn1—N1—C1—C2	−172.62 (12)	C22—N3—C21—C16	−0.1 (2)
C5—N1—C1—C6	−178.14 (13)	C22—N3—C21—C20	179.29 (16)
Mn1—N1—C1—C6	9.13 (17)	C17—C16—C21—N3	−179.84 (15)
N1—C1—C2—C3	0.3 (2)	C15—C16—C21—N3	−0.4 (2)
C6—C1—C2—C3	178.37 (16)	C17—C16—C21—C20	0.8 (2)
C1—C2—C3—C4	−0.6 (3)	C15—C16—C21—C20	−179.84 (16)
C2—C3—C4—C5	0.4 (3)	C19—C20—C21—N3	−179.59 (17)
C1—N1—C5—C4	−0.3 (2)	C19—C20—C21—C16	−0.2 (3)
Mn1—N1—C5—C4	172.30 (11)	C21—N3—C22—C23	179.56 (16)
C1—N1—C5—C7	−179.10 (13)	C21—N3—C22—C27	−0.4 (2)
Mn1—N1—C5—C7	−6.54 (17)	N3—C22—C23—C24	−179.19 (18)
C3—C4—C5—N1	0.0 (2)	C27—C22—C23—C24	0.8 (3)
C3—C4—C5—C7	178.68 (15)	C22—C23—C24—C25	−0.5 (3)
Mn1—O1—C6—O2	−179.12 (17)	C23—C24—C25—C26	−0.1 (4)
Mn1—O1—C6—C1	−1.0 (2)	C24—C25—C26—C27	0.3 (4)
N1—C1—C6—O2	173.11 (17)	N3—C22—C27—C26	179.45 (17)
C2—C1—C6—O2	−5.1 (3)	C23—C22—C27—C26	−0.5 (3)
N1—C1—C6—O1	−5.2 (2)	N3—C22—C27—C15	1.5 (2)
C2—C1—C6—O1	176.59 (16)	C23—C22—C27—C15	−178.46 (16)
Mn1—O3—C7—O4	172.80 (14)	C25—C26—C27—C22	0.0 (3)
Mn1—O3—C7—C5	−7.1 (2)	C25—C26—C27—C15	177.8 (2)
N1—C5—C7—O4	−171.07 (15)	N4—C15—C27—C22	177.46 (17)
C4—C5—C7—O4	10.1 (2)	C16—C15—C27—C22	−2.0 (2)
N1—C5—C7—O3	8.9 (2)	N4—C15—C27—C26	−0.4 (3)
C4—C5—C7—O3	−169.90 (16)	C16—C15—C27—C26	−179.86 (17)
N2—Mn1—O5—C13	0.21 (14)	N6—C28—C29—C34	−176.52 (16)
N1—Mn1—O5—C13	174.50 (15)	C40—C28—C29—C34	4.4 (2)
O7—Mn1—O5—C13	−8.2 (2)	N6—C28—C29—C30	7.2 (3)
O1—Mn1—O5—C13	−112.55 (15)	C40—C28—C29—C30	−171.89 (16)
O3—Mn1—O5—C13	102.30 (15)	C34—C29—C30—C31	2.4 (3)
N2—Mn1—O7—C14	5.78 (16)	C28—C29—C30—C31	178.69 (18)
N1—Mn1—O7—C14	−168.81 (15)	C29—C30—C31—C32	0.6 (3)
O5—Mn1—O7—C14	14.2 (2)	C30—C31—C32—C33	−2.3 (4)
O1—Mn1—O7—C14	116.70 (17)	C31—C32—C33—C34	0.8 (3)
O3—Mn1—O7—C14	−96.39 (17)	C35—N5—C34—C29	−2.7 (3)
N1—Mn1—N2—C12	147.6 (3)	C35—N5—C34—C33	176.34 (17)
O7—Mn1—N2—C12	−4.85 (14)	C30—C29—C34—N5	175.17 (16)
O5—Mn1—N2—C12	−179.90 (15)	C28—C29—C34—N5	−1.3 (2)
O1—Mn1—N2—C12	−95.92 (14)	C30—C29—C34—C33	−3.8 (3)
O3—Mn1—N2—C12	87.60 (14)	C28—C29—C34—C33	179.73 (16)
N1—Mn1—N2—C8	−32.1 (4)	C32—C33—C34—N5	−176.72 (19)
O7—Mn1—N2—C8	175.43 (15)	C32—C33—C34—C29	2.3 (3)
O5—Mn1—N2—C8	0.38 (13)	C34—N5—C35—C36	−176.67 (16)
O1—Mn1—N2—C8	84.37 (14)	C34—N5—C35—C40	3.3 (3)
O3—Mn1—N2—C8	−92.12 (14)	N5—C35—C36—C37	179.03 (17)
C12—N2—C8—C9	0.4 (3)	C40—C35—C36—C37	−1.0 (3)
Mn1—N2—C8—C9	−179.87 (14)	C35—C36—C37—C38	1.5 (3)
C12—N2—C8—C13	179.48 (16)	C36—C37—C38—C39	−0.8 (3)
Mn1—N2—C8—C13	−0.8 (2)	C37—C38—C39—C40	−0.5 (3)
N2—C8—C9—C10	−1.9 (3)	C38—C39—C40—C35	1.0 (3)
C13—C8—C9—C10	179.18 (19)	C38—C39—C40—C28	−179.30 (17)
C8—C9—C10—C11	1.4 (3)	N5—C35—C40—C39	179.74 (16)
C9—C10—C11—C12	0.5 (4)	C36—C35—C40—C39	−0.3 (2)
C8—N2—C12—C11	1.6 (3)	N5—C35—C40—C28	0.0 (2)
Mn1—N2—C12—C11	−178.14 (15)	C36—C35—C40—C28	−179.99 (15)
C8—N2—C12—C14	−176.62 (16)	N6—C28—C40—C39	−2.5 (3)
Mn1—N2—C12—C14	3.7 (2)	C29—C28—C40—C39	176.53 (16)
C10—C11—C12—N2	−2.0 (3)	N6—C28—C40—C35	177.18 (16)
C10—C11—C12—C14	176.0 (2)	C29—C28—C40—C35	−3.8 (2)

### Hydrogen-bond geometry (Å, °)

**Table:**

*D*—H···*A*	*D*—H	H···*A*	*D*···*A*	*D*—H···*A*
N3—H3N···O2	0.88 (2)	1.86 (2)	2.736 (2)	178 (2)
N4—H4A···O4^i^	0.85 (3)	2.22 (3)	2.994 (2)	152 (3)
N4—H4B···O10^ii^	0.93 (3)	2.01 (3)	2.884 (3)	156 (2)
N5—H5N···O11	0.84 (3)	1.87 (3)	2.706 (2)	171 (3)
N6—H6A···O6	0.90 (2)	1.92 (3)	2.801 (2)	164 (2)
N6—H6B···O1^iii^	0.89 (3)	2.20 (3)	3.029 (2)	155 (3)
O9—H9A···O4	0.94 (3)	1.89 (3)	2.815 (2)	171 (3)
O9—H9B···O4^iv^	0.93 (3)	1.93 (3)	2.851 (3)	172 (3)
O10—H10A···O3^v^	0.92 (2)	2.02 (2)	2.926 (2)	168 (3)
O10—H10B···O8	0.92 (3)	1.89 (3)	2.805 (3)	175 (4)
O11—H11A···O6^vi^	0.92 (3)	1.88 (3)	2.790 (3)	170 (4)
O11—H11B···O9^vii^	0.92 (3)	1.85 (3)	2.756 (3)	169 (2)

Symmetry codes: (i) *x*−1, *y*, *z*−1; (ii) −*x*+1, −*y*+1, −*z*; (iii) −*x*+1, −*y*+1, −*z*+1; (iv) −*x*+2, −*y*, −*z*+1; (v) −*x*+2, −*y*+1, −*z*+1; (vi) −*x*+1, −*y*+1, −*z*+2; (vii) *x*, *y*+1, *z*+1.
